# Infectious Laryngotracheitis Virus and Avian Metapneumovirus: A Comprehensive Review

**DOI:** 10.3390/pathogens14010055

**Published:** 2025-01-10

**Authors:** Jongsuk Mo, Jongseo Mo

**Affiliations:** 1 Exotic and Emerging Avian Disease Research Unit, U.S. National Poultry Research Center, Agricultural Research Service, United States Department of Agriculture (USDA), Athens, GA 30605, USA; zelraid55@avinext.co.kr; 2 College of Pharmacy, Yeungnam University, Gyeongsan-si 38541, Gyeongsangbuk-do, Republic of Korea

**Keywords:** infectious laryngotracheitis virus, avian metapneumovirus, herpesvirus, pneumovirus

## Abstract

Respiratory avian viral diseases significantly impact the world poultry sector, leading to notable economic losses. The highly contagious DNA virus, infectious laryngotracheitis virus, and the RNA virus, avian metapneumovirus, are well known for their prevalent effects on avian respiratory systems. The infectious laryngotracheitis virus (ILTV), stemming from the *Herpesviridae* family, manifests as an upper respiratory disease within birds. Characterized by acute respiratory signs, it sporadically emerges worldwide, presenting a persistent threat to poultry health. Avian metapneumovirus (aMPV), belonging to the *Pneumoviridae* family is identified as the cause behind severe rhinotracheitis in turkeys and swollen head syndrome in chickens. This disease can lead to heightened mortality rates, especially when coupled with secondary bacterial infections. This review offers a comprehensive analysis and understanding of the general properties of these specific avian respiratory viruses, control measures, and their global status.

## 1. Introduction

Respiratory avian viral diseases have significantly and negatively impacted the global poultry industry for decades. Such diseases can cause profound implications within densely packed flocks, as the etiologic agents use the respiratory tract as the primary route of infection, leading to prompt and wide dissemination of the virus. Due to the nature of these viruses, they are highly prevalent and can express various levels of morbidity and mortality, leading to performance and especially financial loss due to costly intervention measures. Among the primary causative agents of these diseases, avian influenza virus (AIV), Newcastle disease virus (NDV), and infectious bronchitis virus (IBV) are mainly focused on because of their high mutation rates, high mortality, and prevalence [1,2,3]. Avian influenza is often at the forefront of global discussions due to its zoonotic features, the possibility of interspecies transmission to humans, and pandemic potential. On the other hand, other respiratory viruses, such as infectious laryngotracheitis virus (ILTV) and avian metapneumovirus (aMPV), tend to gain less focus despite their capability of causing significant economic losses and have greater or equal global impacts as other prominent avian viral pathogens. Nonetheless, both viruses are globally distributed, with new subtypes and cases arising in recent years and challenges unique to each virus.

Infectious laryngotracheitis (ILT) is a significant economic burden on the global poultry industry. Infection can reduce egg production, causing weight loss and enhancing bird susceptibility to other respiratory pathogens [4,5,6]. First reported in 1925 [7], the causative agent of ILT is the infectious laryngotracheitis virus (ILTV), a member of the *Herpesviridae* family, specifically within the *Alphaherpesvirinae* subfamily, *iltovirus* genus, and identified as species *Gallid alphaherpesvirus-1* which was formally known as *Gallid herpesvirus-1* [8,9,10]. ILTV primarily affects galliform birds, demonstrating a highly selective host range [11]. While chickens are the most common hosts, the virus has also been isolated from pheasants, peafowl, and, under experimental conditions, turkeys [5,12,13]. The transmission of ILTV occurs not only directly but can also proceed indirectly via fomites, including contaminated equipment, clothing, and trucks, and through improper disposal of manure and infectious litter [14].

Avian metapneumoviruses (aMPVs), classified within the *Pneumoviridae* family of the genus *Metapneumovirus*, are traditionally distinguished into four subgroups (A, B, C, D) based on their sequence analysis and virus neutralization patterns [15]. Initially identified in South Africa during the 1970s, aMPV was believed to infect only turkeys, with chicken infections later linked to swollen head syndrome (SHS) [16,17,18]. The virus worsens secondary bacterial infections, increasing mortality rates, notably with *Ornithobacterium rhinotracheale* (ORT) and *Mycoplasma gallisepticum* (MG) [19,20]. Research has shown that aMPV can enhance ORT’s ability to adhere to and colonize the epithelial cells of the turbinate and trachea, indicating a synergistic relationship between these two pathogens [21]. Despite some level of viral replication in the lungs, aMPV primarily replicates briefly in the upper respiratory tract. Studies have demonstrated that the virus can replicate significantly in turkeys in nasal and sinus tissues for up to 10 days post-inoculation without spreading to other tissues [22,23]. Although mortality rates rarely exceed 2% in chickens, morbidity can affect up to 10% of the population [24]. In addition to causing significant morbidity, the disease is also known to affect egg production, leading to decreased egg quality [25]. Fortunately, vertical transmission in domestic poultry seems relatively uncommon, as only a few cases have been reported, and no viable viruses were isolated from offspring, suggesting that vertical transmission would unlikely play a significant role in virus dissemination [26,27]. AMPV caused USD 15 million in economic losses alone in the United States from 1997 to 2000, further emphasizing the importance of disease control [28]. Like many other RNA viruses, aMPV is known for its heterogeneity, with various subtypes of the strain being distributed and circulating worldwide, primarily by migratory wild birds. This review will focus on these two critical yet frequently overshadowed respiratory viral pathogens.

## 2. The Viral Structure and Classification of ILTV and aMPV

**Figure 1:**
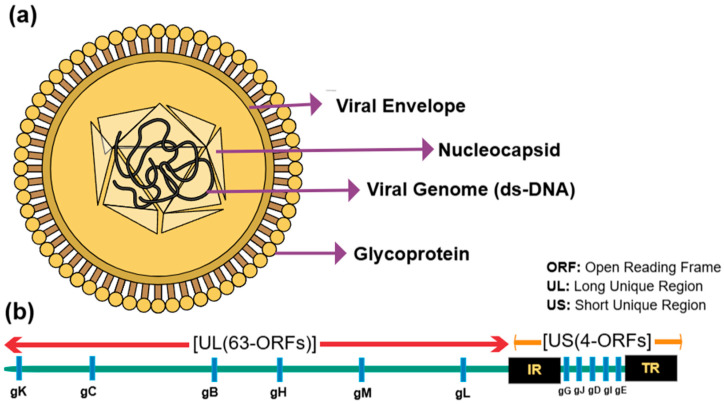
The viral structure of ILTV. (**a**) A schematic diagram of ILTV. (**b**) The genome of ILTV. (Created in https://mindthegraph.com/ (16 September 2024)).

Although ILTV and aMPV are recognized as major respiratory viral pathogens in poultry, they are distinct in several aspects. ILTV shares a similar icosahedral morphology to the herpes simplex virus-1 (HSV-1), featuring a hexagonal nucleocapsid that encapsulates its double-stranded DNA. This nucleocapsid, with an 80–100 nm diameter, comprises 162 elongated and hollow capsomeres [29]. The genome spans approximately 150 kilobase pairs (kb). It includes 79 predicted open reading frames (ORFs) alongside long and short unique regions (UL, US) that are bordered by inverted repeats (internal repeat (IR), terminal repeat (TR)) adjacent to the US region [30,31] (Figure 1). Notably, the genome encompasses two specific gene clusters: one of five ORFs (A-E) located between UL45 and UL22 [32] near the genome’s 5′ end and the UL0, UL(-1) cluster near the 3′ end [11]. In a knockout study, deleting the UL(-1) genes prevented ILTV from replicating in susceptible cell lines, suggesting its crucial role in viral replication [33]. Unlike other *alphaherpesviruses*, which carry only one or two origins of replication (Ori), ILTV carries three Ori of replication: the OriL located in the UL regions and the OriS located at the IR and TR regions [34]. The nucleocapsid is encased in an irregular envelope measuring 195–350 nm in diameter and is adorned with viral glycoproteins on its surface [29,35,36] (Figure 1). Given the genetic and protein similarities between ILTV and HSV-1, the nomenclature for many ORFs and proteins in ILTV has been adopted from HSV-1 [37]. Like other *alphaherpesviruses*, these surface glycoproteins play critical roles in virus mediation, egress, and eliciting immune responses [38]. Historically, five major envelope glycoproteins—gB, gC, gD, gK, and gX—and a unique glycoprotein, gp60, were identified as primary antigens and extensively studied [39,40]. Several glycoproteins (gB, gC, gD, gE, gG, gH, gI, gJ, gK, gL, gM, gN) and their corresponding ORFs have been currently recognized [41]. However, not all their functions and interactions are fully understood [42].

**Figure 2:**
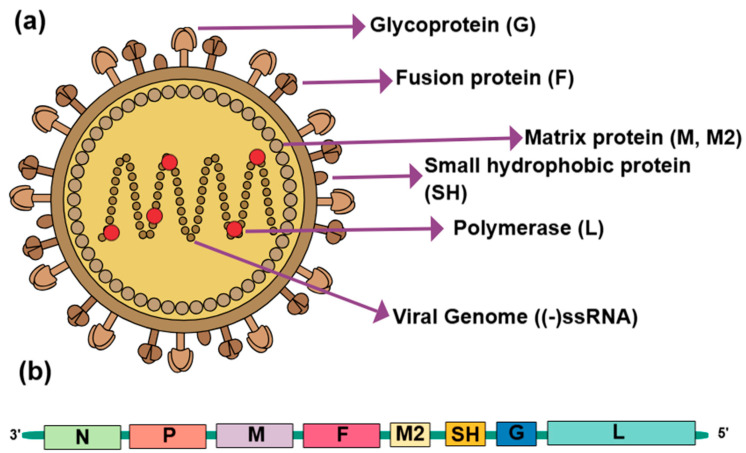
The viral structure of aMPV. (**a**) A schematic diagram of aMPV. (**b**) The genome of aMPV; N: Nucleoprotein, P: Phosphoprotein, M: Matrix protein, F: Fusion protein, M2: Second matrix protein, SH: Small hydrophobic protein, G: Attachment protein, L: Large polymerase protein (Created in https://mindthegraph.com/ (16 September 2024)).

Apart from the DNA virus ILTV, aMPV is an enveloped RNA virus with a negative-sense, non-segmented RNA genome. It shares structural characteristics with members of the *Paramyxoviridae* family. [43]. Its molecular structure showcases a pleomorphic, spherical form with sizes ranging from 50 nm to 200 nm, typically around 150 nm in diameter, and presents a predominantly filamentous appearance [44,45]. Within the virus, the helical nucleocapsid, encased by the matrix (M) protein layer, houses the 13 kb RNA genome. The genomic structure of aMPV encodes eight genes flanked by the 3′ leader and 5′ trailer regions (3′-N-P-M-F-M2-SH-G-L), which are the nucleoprotein (N), phosphoprotein (P), matrix protein (M), fusion protein (F), second matrix protein (M2), small hydrophobic protein (SH), attachment protein (G), and the large polymerase protein (L) [46] (Figure 2). Among them, the most heterogeneous gene in terms of genetic variability is the G gene, which encodes the G protein and is the main target for virus identification and genetic subtyping. This nucleocapsid is notably smaller than paramyxoviruses, such as the Newcastle disease virus (NDV). The lipid envelope of aMPV is embedded with three viral glycoproteins essential for its lifecycle: the G protein, which facilitates host cell attachment; the F; and the SH protein [47]. The F and G proteins form spikes, ranging from 10 to 14 nm in length, on the virus’s surface and are crucial for interacting with the internal side of the viral membrane’s matrix protein layer. For viral entry into a host cell, the G proteins engage with heparin-like or glycosaminoglycan receptors on the cell surface, mediating attachment [48,49,50]. Following this initial attachment, the F protein triggers fusion between the viral envelope and the host cell membrane through a pH-independent mechanism. This fusion facilitates the unpacking of viral components within the cytoplasm, setting the stage for subsequent viral replication. The G protein of aMPV stands out due to its unique sequence and structural properties, differing significantly from the hemagglutinin-neuraminidase (HN) attachment proteins found in other paramyxoviruses [51]. Despite the differences in the G protein, the F proteins of aMPV and other paramyxoviruses share structural similarities despite their sequence heterology [52,53,54]. In the processing of the precursor F0 protein into its active form, it is cleaved into two disulfide-linked subunits, F1 and F2, a mechanism consistent with other paramyxoviruses, mediated by host cell proteases like Furin [55,56]. This cleavage is critical for the virus to fuse with host cell membranes, a crucial step in viral entry.

The M2 gene encodes two overlapping proteins, M2-1 and M2-2, which are involved in the virus’s replication process. Although the function of these proteins is not fully understood, the zinc-binding ability of the M2-1 protein is thought to be involved in regulating viral mRNA transcription and enhancing pathogenesis, while the M2-2 protein functions as a negative regulator of RNA transcription. Some evidence exists for other viruses in the same group suggesting that the M2-2 protein could completely inhibit viral RNA replication [57,58]. The SH protein is an accessory protein commonly found in pneumoviruses and categorized as a type 2 integral membrane glycoprotein [59]. Although the function of this protein is not well known, it is thought to aid in the viral entry process by increasing the membrane permeability of the target cells, inhibiting host immune signaling pathways, and helping the virus evade the host’s immunity [60]. However, based on gene knockout studies, the SH protein does not seem essential for viral replication, as its absence did not significantly affect the fitness of the virus in both in vitro and in vivo settings regarding replication [61,62].

On the other hand, like most DNA viruses, ILTV’s surface proteins are more diverse and numerous than those of aMPV. ILTV glycoprotein B (gB), gC, gG, gJ, gM, and gN have explicitly been identified at the protein level, demonstrating their presence and roles in the viral structure [11,63,64,65,66]. Research has shown that gG, gJ, gM, and gN are not critical for the virus’s ability to replicate in cell cultures [67,68], suggesting alternative functions outside primary viral replication. A particularly intriguing aspect of ILTV’s biology is the attachment mechanism of its glycoproteins B (gB) and C (gC) to host cells. Unlike other *alphaherpesviruses*, sequence analysis has revealed that these ILTV glycoproteins lack the consensus motifs for heparin binding. This finding indicates that ILTV relies on a heparin-independent method for host cell attachment, significantly different from the mechanisms observed in other viruses within the same family [64]. Further comparison of the ILTV gC protein with other *alphaherpesviruses* highlights a notable difference: the absence of arginine and lysine-rich sequences. This deficiency leads to the deletion of approximately 100 amino acids in ILTV gC, resulting in a protein significantly shorter than other gC homologs and lacking the positively charged region in the ectodomain typically required for interaction with heparin [64]. The implications of these structural and functional differences in ILTV’s glycoproteins are profound, especially considering the virus’s host specificity. The absence of heparin-binding features in gB and gC suggests that ILTV’s entry into host cells—and, by extension, its narrow host range—may be determined by relying on alternative cell surface receptors alongside heparin-independent entry pathways. This adaptation could play a crucial role in defining the susceptibility of host species to ILTV infection. Glycoproteins H (gH) and gL are heterodimeric complexes that participate in herpesviruses’ core fusion machinery. They are also the primary targets for virus-neutralizing antibodies [69]. Viral entry mediated by the gH/gL complex is another method of heparin-independent entry. Functional homologs of gL co-processed in a complex with gH are commonly discovered in most herpesviruses, including ILTV. N-terminal signal sequences, N-glycosylation sites, and two cysteine residues are also present in gL of ILTV, suggesting a similar function to other herpesviruses [32]. Glycoprotein H is a type I integral membrane protein needed for the viral incorporation of gL, which lacks the membrane anchor [70], thus forming the gH/gL complex.

Glycoprotein D (gD) is also a major immunogenic glycoprotein that plays a vital role in cell receptor binding. Like other herpesviruses, gD of ILTV acts as a receptor for virus binding to target cells and triggers entry through receptor-mediated displacement of its C-terminal region [71]. During the fusion process of the viral and cell membranes, gD must interact with susceptible cell receptors such as herpesvirus entry mediators (HVEMs) and nectins to achieve successful viral entry. An HVEM is a tumor necrosis factor receptor (TNFR)-like receptor found mainly on the surface of immune cells, while nectins are cell adhesion molecules commonly expressed in neural or epithelial cells [72,73]. Viral entry using nectins may be related to the virus’s neuro-invasive property when infecting the host’s trigeminal ganglion [74,75]. Glycoprotein D is also a well-known target for inducing neutralizing antibodies [73] and has been the main target immunogen for recombinant vaccine development [76].

Although viral recombination of ILTV as a DNA virus is not as dynamic as other highly mutative avian RNA viruses, recombinants still emerge based on co-infections within the same host, especially between attenuated vaccine and wild-type strains [77]. Events of recombination between ILTV wild-type strains and vaccine strains are well documented, and further evidence was revealed in a recent study based on an analysis of the complete genome of ILTV [78]. Also, regarding recombinational breakpoints within the viral genome, single-nucleotide polymorphisms (SNPs) were discovered in the US5, US6, US7, US8, US9, and UL43 and UL47 genes, confirming potential high recombination sites within the viral genome [79]. Genetic mutations in some regions of the ILTV genome are associated with strain virulence. For instance, nonstructural genes such as UL28, UL5, and ICP4 genes are believed to be the determinants of the virulence or attenuation of ILTV [80] which encode the DNA packaging protein [81], the helicase-primase [82], and the major transcriptional regulatory protein, respectively [83,84]. Nucleotide changes in the thymidine kinase (TK) gene also seem to increase or decrease virulence [85,86]. Moreover, single nucleotide changes in the open reading frame (ORF) of C, gB, UL39 (ribonucleotide reductase), and UL41 (virion host shutoff protein) are known to be related to events of live ILTV vaccines regaining virulence [87]. The UL0 gene encodes an abundantly expressed protein that accumulates in the nucleus of ILTV-susceptible cells and is known to be directly involved in viral replication and virulence [88]. One of the well-known and fully proven virulence factors in ILTV is the gG gene [67]. In contrast, the UL50 gene that encodes the deoxyuridine triphosphatase (dUTPase) required for dTTP synthesis was discovered to be a non-virulence factor [89]. Such genes can serve as targets for live attenuation of the ILTV virus to produce deletion mutant strains that can be used as vaccines. Since live attenuated vaccines were among the first developed to control ILTV, this field has a long history of identifying the roles of specific genes and performing knockout studies to establish a safe and stable vaccine that can be used in the field.

Like other RNA viruses, aMPV is more prone to mutations compared to other DNA viruses such as ILTV and is classified into four subtypes (A, B, C, D) based on nucleotide and amino acid sequence analyses, particularly of the G protein, and by using neutralization assays involving monoclonal antibodies [90,91]. On the other hand, ILTV classification is usually based on restriction fragment polymorphism (RFLP) of genome regions to characterize field isolates [92,93,94]. Due to their more conservative genetic homogeneity and similarities in the genome compared to aMPV, classification is crucial once live attenuated vaccine strains are introduced in the field [95].

Regarding the classification of ILTV, RFLP of polymerase chain reaction products (PCR-RLFP) greatly facilitated the identification of ILTV field isolates [94]. Nowadays, ILTV field isolates are differentiated based on the ICP4 region or the complete genome [80,96,97]. The ICP4 gene has been utilized intensively as a target to identify and group ILTV in various regions worldwide due to its highly conserved regions with fewer genetic similarities [85,97,98,99,100]. Moreover, it was proven that partial analysis of the ICP4 gene can provide comparable results to conventional PCR-RFLP analysis [101]. Recent cases show that the analysis of ICP4 was successful in differentiating live attenuated vaccine strains and field ILTV isolates, with distinctive differences in nucleotide sequences [99,102]. In addition, three clades of ILTV have been proposed based on complete genome sequencing [6]; Clade I: strains originating from the US chicken embryo-originated (CEO) vaccine Hudson strain, the European CEO vaccine Serva strain, and the Australian CEO-like ACC78 (CL8) virulent strain; Clade II: US embryonic tissue culture origin (TCO) strains and virulent US strains (USDA reference and 81658); and Clade III: an Australian vaccine (SA2 and A20) and virulent strains (CSW-1 and VI-99), and one virulent strain from China (LJS09).

## 3. The Clinical Manifestations of ILTV and aMPV

Despite both being major respiratory viral pathogens in avians, while similarities between their clinical signs exist, there are some significant differences regarding their pathogenesis and disease progression. The clinical signs of ILTV infection typically emerge between six and twelve days following natural exposure to the virus [4]. Signs can vary from hemorrhagic conjunctivitis, watery eyes, and nasal discharge to respiratory difficulties, including rales, gasping, and the expulsion of blood-stained mucus, especially in severe disease cases [6,87]. Gross lesions of the disease are typically confined to the upper respiratory tract and sinuses [103]. Based on the anatomy of the upper respiratory tract, ILTV is likely to infect cells within the nasal cavity, conjunctiva, and harderian glands in the early stages of infection. These structures contain immune tissues that can act as first-line barriers against the virus [104] and can determine the overall outcome of infection. As the disease progresses, the virus can further infect the tracheal basal cells and conjunctival mucosa, leading to rapid transmission within the flock [105]. Existing evidence also suggests a systemic distribution within the host, as ILTV was detected by quantitative real-time polymerase chain reaction (qPCR) in multiple organs outside the upper respiratory tract, from the brain, lung, and heart to the liver, kidney, and bursa of Fabricius in experimentally infected birds [106]. The exhibition of tissue tropism other than in the respiratory tract may be somewhat related to the severity of the disease.

**Figure 3:**
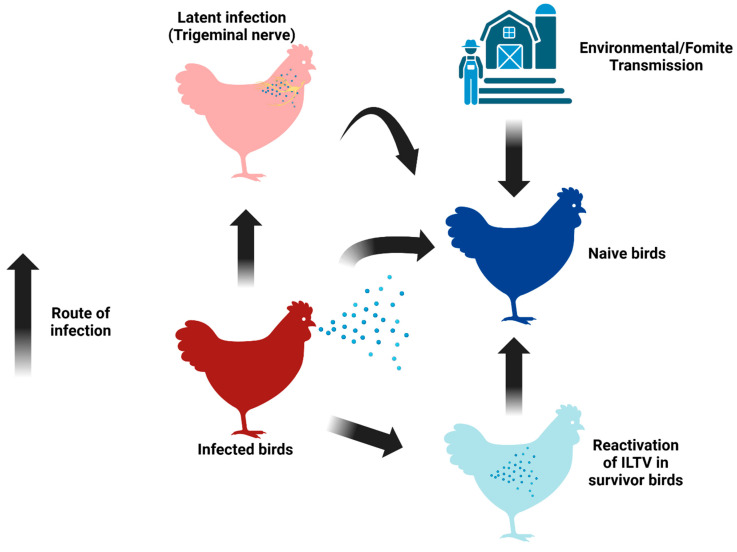
The infection route and dissemination patterns of ILTV (created in https://BioRender.com (28 September 2024)).

Multiple factors, such as the virulence of the strain and its concentration in the environment, alongside co-infections with other respiratory pathogens, can influence the severity of ILTV infection. In some instances, particularly severe outbreaks of the disease have led to morbidity rates reaching up to 100% and mortality rates soaring to 70% [95]. Following infection, the virus undergoes replication predominantly within the trachea, initiated by ingestion through the upper respiratory and ocular pathways [36,107]. Peak viral replication in the tracheal epithelium is observed from two to five days post-infection [108], with low levels of ILTV occasionally detectable up to ten days post-infection. However, active replication is generally restricted to the first week following infection [109,110]. Like other herpesviruses, ILTV can become a latent infection, which can be reactivated and shed in response to various stress factors (Figure 3), a distinctive feature compared to aMPV. The trigeminal ganglion (TRG) is identified as the primary site for viral latency during the lytic phase of ILTV infection [40]. Stress factors that can trigger the virus’s reactivation include vaccination, the onset of laying, relocation, etc. [10]. More problematically, latent or reactivated ILTV carriers are also well-known transmission sources apart from direct virus dissemination from active virus-shedding birds. Other external viral sources include contaminated dust, litter, and fomites (Figure 3).

Compared to ILTV, the host range of aMPV is rather broad. It affects various avian species, while its natural hosts are turkeys and chickens. Evidence of virus infection has been discovered in a variety of wild birds, such as wild geese [111,112], pheasants [113], guinea fowl [114], waterfowl [115], mallard ducks [116], crows and coots [117], and many others [118,119]. Increasing evidence of the prevalence of aMPV in wild birds suggests a strong possibility of them being reservoirs and inter-regional carriers of this virus, capable of being introduced into domestic poultry. Moreover, the species of the principal hosts (chickens, turkeys, ducks) infected in past cases differ depending on the subtype, as the majority of aMPV-A, B, and C were detected in chickens and turkeys, Eurasian subtype C lineage in ducks, and subtype D in turkeys [120]. The virus’s ability to replicate and transmit depends on how well the virus is adapted to a specific group of hosts. Experimental evidence suggests that aMPV can quickly modify its genome to readily adapt to a new host [121].

**Figure 4:**
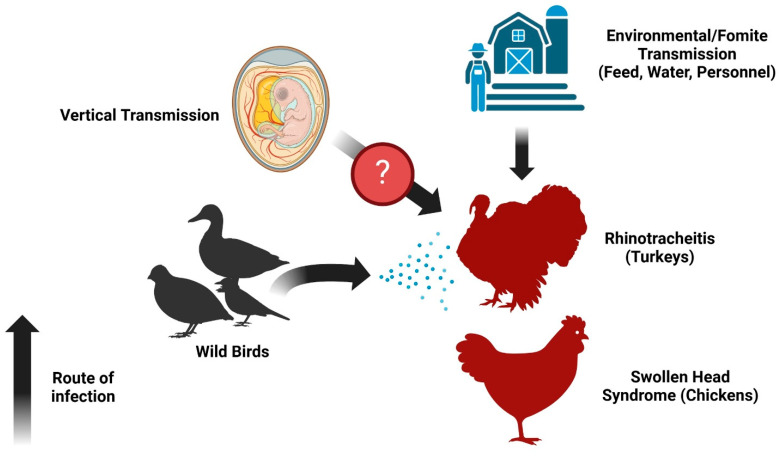
The infection route and dissemination patterns of aMPV (created in https://BioRender.com (27 September 2024)).

AMPV causes rhinotracheitis in turkeys (Figure 4). Clinical signs in infected turkeys typically appear after an incubation period of 3–7 days. Infected birds generally exhibit respiratory signs such as tracheal rales, watery nasal discharges, coughing, and signs around the eye such as periorbital edema and conjunctivitis [24,122]. The clinical signs around the sinus area are attributed to the accumulation of mucoid fluid following infection [123]. One of the notable signs in chickens is swollen head syndrome (SHS), which is associated with head and facial edema resulting from the accumulation of mucous exudates in the subcutaneous tissue [124]. The development of SHS is typically associated with secondary bacterial infections such as *Escherichia coli* (*E.coli*), followed by initial aMPV infections [125]. Environmental conditions are also linked to the severity of SHS, such as high concentrations of ammonia and dust, as well as improper ventilation and hygiene [126]. Regarding egg production, it was found that aMPV can replicate in the epithelial cells of the oviduct in turkey layers, leading to lower eggshell quality and a decline of 10–40% in egg production, causing massive economic losses [24]. Additionally, a disease caused by aMPV can get worse with co-infections with other pathogens, notably *Mycoplasma gallisepticum* (MG) [126], and with other respiratory viral diseases such as Newcastle disease virus (NDV) [127]. Vertical transmission of aMPV from layers to progeny may seem possible, although there are few cases. In an experimental trial of infecting turkey layers with aMPV subtype C, the presence of the virus was confirmed on the eggshell and the embryo [128]. However, despite evidence of vertical transmission, it has not yet been fully proven [118,129] (Figure 4).

ILTV’s outbreaks and field infections are somewhat unique compared to aMPV, in which the introduction of aMPV to domestic birds is mainly thought to have directly originated from wild-type strains by wild birds. Genetic analysis based on PCR-RFLP analysis has consistently shown that most ILTV isolates from commercial poultry within the US bear close relation to the strains used in commercial chicken embryo-originated or CEO vaccines [130,131,132]. This relationship appears stable over time, as indicated by a more recent study employing the same genotyping technique, which found no significant changes in the genetic makeup of these isolates over the years [94]. Similar findings have been observed in studies conducted outside the US, where field isolates of ILTV were also traced back to vaccine strains, confirming that this is not a unique phenomenon [133,134]. What is more alarming is that these CEO vaccine-related ILTV recombinant strains are beginning to appear in regions where ILTV vaccination is prohibited. In 2024, it was reported from an epidemiological study in Switzerland that two-thirds of the analyzed ILTV strains were related to CEO vaccines in a country where vaccination is de facto prohibited [135]. This likely was a result of virus spillovers from neighboring countries.

## 4. The Global Distribution of ILTV and aMPV

**Figure 5:**
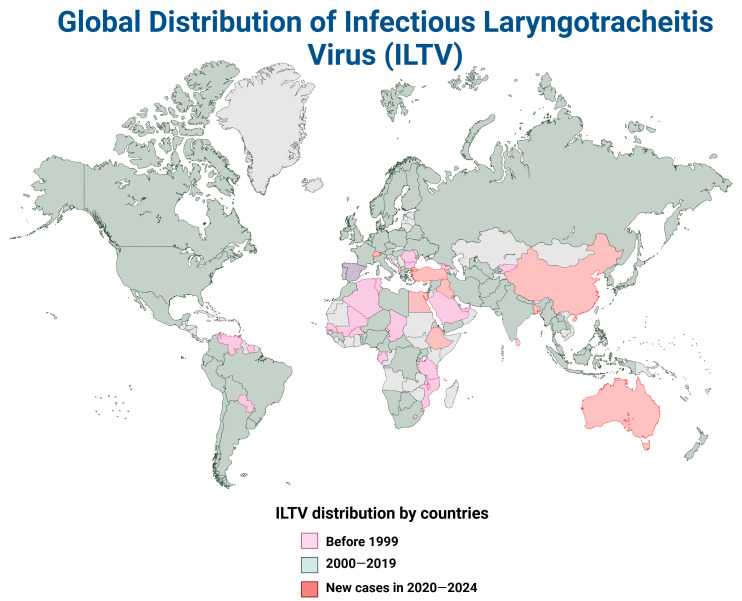
The global distribution of ILTV (data from Menendez et al. [6] included) (created in https://BioRender.com (29 August 2024)).

Like other prominent avian respiratory viral pathogens, ILTV and aMPV are also prevalent worldwide, although ILTV has a more extended history of global distribution. ILTV has been prevalent for decades, covering nearly every continent, from Asia [136,137,138,139] to Europe [41,135,140], Africa [141], North and South America [94,101,142,143], and Oceanian regions [6,10]. Recent outbreaks include areas in Australia [144,145], Iraq [136], and Egypt [146], where the circulation of recombinant strains with increased virulence was confirmed (Figure 5). Further cases of ILTV circulation in the region were reported in Switzerland [135], Turkey [147,148], Bangladesh [149], Ethiopia [150], and China [151] between 2020 and 2024. Historically, ILTV outbreaks were reported in over 100 nations between 2000 and 2013 [6]. In the United States, 88 cases of ILTV were confirmed in California alone during the period 2007–2017 [97], although most of the cases involved mild clinical forms of ILT. Nonetheless, ILTV remains a severe threat to the world poultry sector. The morbidity and mortality of reported cases largely depend on the virulence of the strain [67] circulating in the region alongside concurrent infection with other respiratory bacterial and viral pathogens.

**Figure 6:**
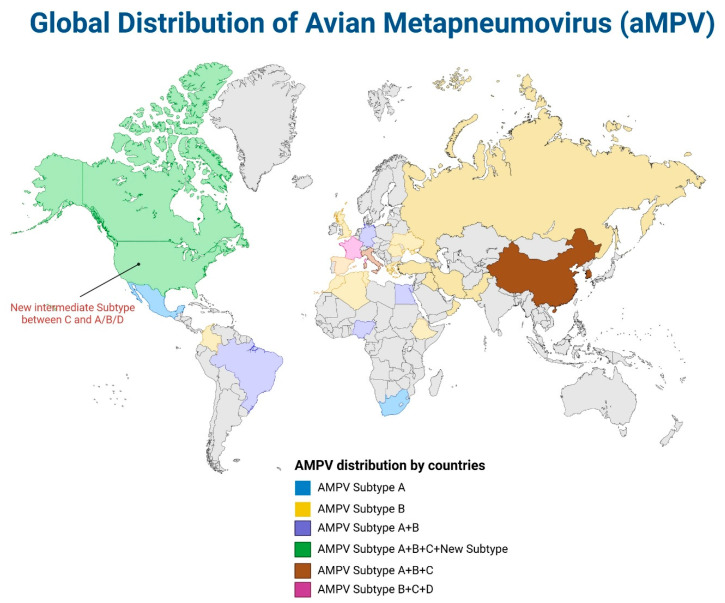
The global distribution of aMPV (created in https://BioRender.com (29 August 2024).

Regarding aMPV, subtypes A and B are the most common, with a significant presence on many continents, including Asia, Africa, Europe, and South America. Subtype C is categorized into two lineages, North American and Eurasian, with the North American lineage being the first to be identified in the mid-1990s in the US and the latter in France and China. Subtype D, considered a relatively minor subtype, was first identified in 2000 and only confirmed in France [15]. In the past, subtypes A and B were geographically confined in continental Europe and the UK [152,153], subtype C in North America, and subtype D in France [15,115] (Figure 6). However, recently, in the US, starting from the fall season of 2024, the spread of subtypes A and B was reported in California and Virginia, respectively, with subtype A being dominant in the western states and subtype B in the eastern states [154,155]. Even though these subtypes have only been circulating for a short time, as turkeys are known to be more susceptible to the disease, the spread of aMPV may have more devastating effects on the US poultry industry because domestic turkey production is significantly higher than in other countries [156,157]. The rapid re-emergence of subtypes A and B in the U.S. is interesting because aMPV has not been present in the U.S. for an extended period since the early 2000s. The genetic sequence of the newly identified aMPV subtype A was first revealed from a sick turkey in California in 2024 [158]. The new strain was phylogenetically grouped with Mexican aMPV strains from 2022, indicating possibilities of a transborder spillover between the two countries [159]. The increasing prevalence of aMPV cases is a global phenomenon. In 2022, cases involving aMPV subtype B causing SHS in broilers in Iraq were first reported [160]. Subtype B was reported in Brazil [161] and Tunisia [162] in 2023. Subtype C was also isolated from China in the same year [163]. In 2024, an epidemiological survey of aMPV in Morocco revealed that subtype B has been prevalent in broiler farms, indicating the wide spread of this subtype nationwide [164]. Subtype C was also recently reported to be widespread in wild birds in Italy [120], suggesting their role as carriers or reservoirs. Also, in the same year, 2024, the seroprevalence of aMPV was reported in pullet and layer hens in Thailand, highlighting the endemicity of the virus [165]. The circulation of subtype B was also confirmed in Columbia in 2024 [129].

Besides the four former subtypes, new aMPV strains forming distinctive subtypes have recently been reported from wild birds involving North American aquatic birds and monk parakeets [166,167] (Figure 5). The first member of a newly emerging subtype was identified based on whole-genome sequencing of a strain isolated from a sick monk parakeet residing in a captive breeding center. This new representing aMPV strain only shared 61–66% similarities with the former subtypes [167]. In another case, a new aMPV strain was discovered in American herring gulls and great black gulls, which leads to a new intermediate subgroup between aMPV-C and the other A, B, and D subtypes [121]. Whether the discovery of these new strains is the start of a newly emerging subtype group warrants further investigation. Such worldwide distribution emphasizes the ongoing global impact of various subtypes of aMPV, including potentially new groups. Both ILTV and aMPV significantly threaten poultry industries worldwide and affect domestic birds. Therefore, the differentiation of their subtypes is crucial and aids in understanding the epidemiological landscape and controlling infections caused by these viruses.

## 5. Vaccine Development for ILTV and aMPV

Vaccination, strict biosecurity measures, and rapid diagnosis are cornerstones in the control of ILTV and aMPV. Vaccination programs for ILTV are predominantly administered in regions where ILTV is endemic, given that live attenuated vaccines have the potential to remain in a latent state within the sensory ganglia or trigeminal ganglion of birds, leading to the creation of long-term carriers or reservoirs [36,95,168,169] (Figure 3). Despite this, vaccination during an outbreak can effectively curb the spread of the virus and reduce the disease’s duration. The primary vaccines administered against ILTV include live attenuated and recombinant viral-vectored vaccines. Historically, two live attenuated ILT vaccines have been approved and utilized: those of chicken embryo origin (CEO) [170] and those developed from embryonic tissue culture origin (TCO) [171]. Although these vaccines are generally effective, there are concerns regarding their use, particularly with CEO vaccines, which in some instances have resulted in reduced performance due to potential residual virulence. This residual virulence may even escalate through animal passage, posing a risk of transmission from vaccinated to unvaccinated birds and the possibility of reverting to more virulent forms after multiple passages within a flock. Outbreaks caused by virulently reverted ILTV strains derived from CEO vaccines have been reported globally, affecting regions across North and South America, Australia, and Europe [6]. Conversely, outbreaks linked to TCO vaccine strains are comparatively rare [80].

The origins of CEO vaccine strains trace back to virulent US field strains from the 1950s and 1960s which underwent attenuation through serial passages [87]. Administration methods for CEO vaccines include drinking water, coarse spray, and eye drops, while TCO vaccines are administered explicitly via eye drops [94,171]. The CEO vaccine is noted for providing superior protection compared to the TCO vaccine, particularly in clearing the challenge virus and the higher replication rates of the vaccine strain. Nonetheless, CEO strains reverted to virulent strains can induce more severe clinical signs than TCO revertant strains at comparable passage levels, highlighting the complexity of vaccine choice and administration in managing ILTV.

Vaccines for aMPV have been available in Europe since the late 1980s, offering means to control the disease effectively [172,173]. Despite the wide availability of these vaccines, disease outbreaks in vaccinated flocks still have occurred, similar to the challenges observed with NDV, indicating events of vaccine failure [174,175]. Early theories suggested that vaccine failure could be attributed to poor vaccination techniques or vaccine strains not matching the subtypes of circulating field strains. While mismatched vaccine seeds contributed to some failures, a more complex issue was revealed, as sometimes protection was not guaranteed even when a vaccine strain matched the target field strain’s subtype [174]. This pointed toward genetic variations within field isolates, particularly in the G protein region, as a potential cause for vaccine escape. For instance, a study genetically analyzing Italian subtype B strains isolated between 1987 and 2007 identified consistent mutations in the G gene region [176]. This led to non-synonymous mutations that altered the amino acid sequence, which could undermine vaccine efficacy. Further analysis revealed that changes in the SH protein were as significant as those observed in the G protein, while other viral genes remained relatively stable. This observation was supported by further vaccination trials and complete genome sequencing data, which correlated significant amino acid changes in the G and SH proteins with reduced vaccine protection. Although its function is yet to be fully understood, the SH protein is an integral membrane protein thought to interact with the host immune system, potentially influencing viral fitness and immunogenicity within the host. A knockout study deleting the SH gene speculated that its absence could affect mounting immune responses [177]. Recombinant strains lacking the SH gene displayed markedly reduced fitness in both in vitro and in vivo experiments [177]. These findings highlight the SH protein’s potential role in aMPV’s pathogenesis and vaccine interaction, emphasizing the need for continuous monitoring of viral evolution to enhance vaccine design and efficacy.

Regarding vaccine development trends for ILTV, in response to epidemics linked to CEO vaccine-derived strains, recombinant vaccines based on fowl poxvirus (FPV) or turkey herpesvirus (HVT) as viral vectors have been developed [5]. These include an FPV vector vaccine incorporating glycoprotein B and UL32 genes as immunogens, initially introduced for use in breeders and commercial layers [178]. HVT-vectored vaccines that utilize glycoprotein B, or glycoproteins I and D, as immunogens have also been formulated [5]. The key advantages of using HVT and FPV as vaccine vectors are their inability to transmit within the flock and their low risk of reverting to a virulent form [179,180]. Vector vaccines based on NDV are also popular and have been intensively focused. The Lasota-based NDV strains encoding ILTV glycoproteins have been developed to overcome conventional vaccines’ safety and biosecurity concerns [181,182,183]. The protective efficacy of such NDV-vectored vaccines seemed suitable. Three NDV-vectored vaccines, each expressing gB, gV, and gD of ILTV, were developed and tested [181]. It was found that gD was mostly efficiently expressed on the surface of the NDV envelope, and the gD-encoded vaccine could fully protect against highly virulent ILTV and NDV challenges. Another NDV-vectored vaccine produced after eight serial passages in embryonated chicken eggs (ECEs) that expressed gD of ILTV still maintained its genetic stability, showing its potential as a safe and stable vaccine [182]. NDV-vectored vaccines expressing other glycoproteins besides gD have also turned out to be successful. Recently, in 2023, a recombinant thermostable NDV-vectored vaccine expressing gB provided efficient protection against the ILTV challenge, significantly reducing viral shedding [183]. Since NDV vaccines based on thermostable avirulent stains have been proven to be advantageous as they can be administered through the environment (water, sprays, feed, etc. [184]), using this backbone for developing future ILTV vaccines is not surprising. 

While these viral vector vaccines have shown efficacy in reducing the clinical signs of infection, they are generally less effective than traditional live attenuated CEO/TCO vaccines in minimizing virus shedding. The success of these vector vaccines largely depends on the accuracy of vaccine administration methods; improper application can significantly reduce their effectiveness, resulting in partial protection. Other issues such as technical failures in delivering vaccines to their intended sites (amniotic cavity, embryo muscle) during in ovo vaccination or reductions in vaccine doses due to economic reasons pose significant challenges to such vaccine strategies [14,185,186].

Gene-deletion-based recombinant ILTV vaccines have been extensively explored to create recombinant strains that maintain robust growth without presenting growth defects [187,188]. One of the notable advancements in this area is the development of an ORF C gene-deleted strain (ΔORF C), which demonstrates no impact on the fitness of the recombinant strain in vitro. This strain exhibited protection titers similar to the parental strain and has shown efficacy comparable to TCO vaccines when administered via eye drops [189]. Other gene-deleted mutant recombinant ILTV vaccines also exist. Various attempts to develop safe and stable recombinant ILTV vaccines by deleting specific genes have been made in the past and are still being made. Targets of deletion include genes like gG [67,190], gC [42], UL47 [191], UL0 [88], TK [192], and gJ [68]. Deleting these genes seems reasonable, as they are primarily involved in modulating host immune responses, viral attachment, replication, and virion assembly. Recently, more sophisticatedly designed recombinant ILTV vaccines have been developed using immunoinformatic tools. Several T-cell and B-cell epitopes have been suggested for multiepitope peptide vaccines aiming to bind gB to these candidate epitopes [193]. In a similar study, a multiepitope vaccine against gD of ILTV was also designed using immunoinformatic tools [194]. The advantage of immunogenically tailored vaccines is that they may effectively induce sufficient humoral and cell-mediated responses from the host. As promising as these vaccines are, as they were only validated at the in silico stage, further in vitro and animal studies are imperative for their use in the field.

Vaccination to reduce consistent virus shedding from aMPV-infected flocks is considered a primary objective, as virus shedding can contribute to mutating aMPV field strains. Research indicates that when live aMPV vaccine strains persist in the environment, these strains can revert to a virulent form [175,195]. This reversion and mutation can occur under the selective pressure of immune responses in vaccinated flocks, leading to the emergence and dissemination of mutant strains capable of sustaining their fitness and viability despite the immunological barriers presented by the host. Given this backdrop of vaccine-induced selective pressure and the potential for live vaccines to shed and revert to virulence, there is an ongoing exploration of alternative vaccination strategies. Such types of vaccines include viral-vectored vaccines based on recombinant strains developed from reverse genetics systems (RGSs). Like ILTV vaccines, NDV vectors are also widely used in aMPV vaccine development [196,197,198]. Most of these NDV-vectored vaccines aim to express the G protein, which is responsible for viral attachment to susceptible host cells and is the main target for neutralizing antibodies. However, there were some cases where expression of the G protein alone did not provide complete protection against pathogenic aMPV, suggesting co-expression of other immunogenic proteins [196,198]. A bivalent NDV-vectored vaccine expressing both the G and F proteins was able to provide efficient protection against a virulent aMPV challenge [197]. Despite the main focus on RGS-based vectored vaccines, live attenuated vaccines produced through serial passage in cells are still being developed, with some exhibiting complete protection efficacy [199].

Utilizing RGSs for aMPV vaccines represents a significant advancement in this area. Past research involving RGSs has opened up pathways for developing non-shedding live attenuated vaccines [177,200]. Such vaccines, engineered not to be shed by vaccinated birds into the environment, could mitigate the risk of generating virulent revertants or promoting the evolution of vaccine-resistant strains. However, successfully developing and implementing these vaccines requires a deeper understanding of aMPV genetics and the interactions between viral proteins and the host immune system. Focusing on virus–host interplay is crucial for developing vaccines that are effective in inducing immunity and safe in terms of viral mutation and spread. This approach underscores the need for continuous research and innovation in vaccine technology to address the challenges posed by aMPV and similar pathogens.

Overall, such advancements will lead to the development of safe and effective ILTV and aMPV vaccines, potentially offering new solutions for managing and preventing outbreaks within the poultry industry.

## 6. Conclusions

ILTV and aMPV share key similarities and differences in clinical manifestations and epidemiology. ILTV primarily causes respiratory distress and is typically confined to the upper respiratory tract in chickens. In contrast, aMPV, in addition to respiratory signs, can additionally impact the reproduction system in both turkeys and chickens, leading to reduced egg production. ILTV tends to exhibit higher mortalities, while aMPV generally results in lower mortality rates but causes significant economic losses due to declined performance. Both ILTV and aMPV exhibit species-specific prevalence and seasonal patterns, with ILTV being more common in broilers and aMPV impacting a wider range of avian hosts, including wild birds, particularly waterfowls. ILTV is more prone to fomite transmission, and like other herpesviruses, it can go through a latent stage which complicates control strategies as reactivation can lead to intermittent shedding of live viruses within densely packed flocks. In the case of aMPV, multi-species involvement makes intervention more challenging, requiring a focus on wild bird populations and natural reservoirs. Despite the key differences, both viruses are major respiratory avian pathogens that require vaccination and strict biosecurity as primary control measures.

In conclusion, respiratory viral diseases are common in avians and cause economic loss worldwide, including in the poultry industry. Like most avian viral respiratory pathogens, infectious laryngotracheitis virus and avian metapneumovirus have a long history of causing problems and, to date, still are problematic. They are highly transmissible and can cause high morbidity and mortality within flocks if not appropriately treated. Therefore, early detection of these viruses is crucial so appropriate control measures can be implemented promptly. As there are increasing reports of the re-emergence of these viruses, including new cases from regions that have never been reported before, constant and active surveillance based on new sequencing technology, alongside frequent updates of the genetic information of the newly circulating viruses, is imperative.
