# Correction: Mo, J.; Mo, J. Infectious Laryngotracheitis Virus and Avian Metapneumovirus: A Comprehensive Review. *Pathogens* 2025, *14*, 55

**DOI:** 10.3390/pathogens14050497

**Published:** 2025-05-19

**Authors:** Jongsuk Mo, Jongseo Mo

**Affiliations:** 1 Exotic and Emerging Avian Disease Research Unit, U.S. National Poultry Research Center, Agricultural Research Service, United States Department of Agriculture (USDA), Athens, GA 30605, USA; zelraid55@avinext.co.kr; 2 College of Pharmacy, Yeungnam University, Gyeongsan-si 38541, Gyeongsangbuk-do, Republic of Korea

There was an error in the original publication [1]. The authors requested the removal of the following sentences in paragraph 2 of Section 2. The Viral Structure and Classification of ILTV and aMPV, and the references cited within it. The authors requested this removal because the nonstructural proteins NS1 and NS2 are present in Respiratory Syncytial Virus (RSV), but not in Avian Metapneumovirus (AMPV).

“AMPV also encodes nonstructural proteins NS1 and NS2 that play significant roles in evading the host immune response. These proteins are expressed in higher quantities than other viral proteins and function by inhibiting RNA synthesis and interferon activation, facilitating viral replication and spread within the host [57–59].”

With this correction, the order of some references has been adjusted accordingly. The authors state that the scientific conclusions are unaffected. This correction was approved by the Academic Editor. The original publication has also been updated.
